# The primary preventive effect of sodium–glucose cotransporter inhibitors on chronic kidney disease in patients with type 2 diabetes: a systematic review and meta-analysis

**DOI:** 10.3389/fendo.2026.1761317

**Published:** 2026-02-27

**Authors:** Zhenlin Zhang, Qingxing Xie, Ziwei Ye, Fang Zhang, Jing Li, Yuwei Zhang, Qingguo Lv, Suming Shen, Nanwei Tong

**Affiliations:** Department of Endocrinology and Metabolism, Laboratory for Diabetes and Metabolism Research, West China Hospital, Sichuan University, Chengdu, China

**Keywords:** chronic kidney disease, meta-analysis, prevention, sodium-glucose cotransporter inhibitors, type 2 diabetes

## Abstract

**Aim:**

To assess the efficacy of Sodium–glucose cotransporter inhibitors (SGLTis) in the primary prevention (PP) of chronic kidney disease (CKD) among patients with type 2 diabetes (T2D).

**Method:**

Literature was retrieved from MEDLINE, Embase, and Cochrane databases up to January 1, 2025. Eligible studies included randomized controlled trials (RCTs) or their subgroups and observational studies involving T2D patients without CKD treated with SGLTis for ≥ 1 year, focusing on CKD-related composite outcomes. Seven articles meeting inclusion criteria were included. The hazard ratio (RR) was calculated, and the degree of heterogeneity was assessed. Subsequently, 95% confidence intervals (95% CI) were computed accordingly.

**Result:**

In RCTs, 15,228 eligible participants received SGLTis and 12,736 received placebo. Meta-analysis using random-effects models showed that SGLTis reduced CKD-related composite outcomes by 53% (RR 0.47, 95% CI 0.39, 0.57; *P* < 0.0001) with low heterogeneity (I² = 6%). Observational data also indicated a lower CKD incidence (0.6–0.7%) with SGLTis versus other glucose-lowering therapies in individuals with CKD PP.

**Conclusions:**

SGLTis significantly lower CKD incidence in T2D patients without baseline CKD. Further RCTs are necessary to validate our conclusions.

**Systematic Review Registration:**

https://www.crd.york.ac.uk/PROSPERO/, identifier CRD420251168634.

## 1 Introduction

As the annual prevalence of chronic kidney disease (CKD) continues to increase, CKD has become a significant public health concern that necessitates collaborative prevention and management strategies across nations (1). Although there are multiple etiological factors contributing to CKD, metabolic dysfunction syndrome associated with type 2 diabetes (T2D) has emerged as a predominant global cause of CKD in recent years (2). T2D significantly elevates the risk of end-stage renal disease (ESRD), adverse cardiovascular events, and overall mortality (3). Conversely, CKD significantly increases the risk of adverse prognoses in patients with T2D, thereby establishing a detrimental cycle. Sodium-glucose cotransporter inhibitors (SGLTis) represent an innovative class of oral hypoglycemic agents. Currently available options can be categorized into two main groups: SGLT2is, such as dapagliflozin and empagliflozin, and dual-receptor inhibitors that target both SGLT1 and SGLT2 (SGLT1/2i), including canagliflozin and sotagliflozin. Large-scale prospective randomized controlled trials (RCTs) have demonstrated that these medications can significantly mitigate the risks of cardiovascular events and renal outcomes in patients with T2D who also present with CKD, thus delaying the progression of CKD (4–10).

Despite this, there remains a significant gap in research investigating the impact of SGLTis on the incidence of CKD among individuals with T2D who do not yet have CKD, specifically regarding primary prevention (PP) of CKD. Given that CKD is currently considered an irreversible condition, it is essential to prevent its onset in T2D patients before it manifests in order to effectively address this issue (11). We aim to evaluate the primary preventive effects of SGLTis on CKD in T2D patients through a meta-analysis or systematic review, thereby providing valuable evidence for clinical guidance.

## 2 Research design and methods

The research protocol was registered on International prospective register of systematic reviews (PROSPERO) (identifier CRD420251168634 [available at https://www.crd.york.ac.uk/PROSPERO/view/CRD420251168634]). This systematic review and meta-analysis adhere to the Preferred Reporting Items for Systematic Reviews and Meta-Analysis (PRISMA) guidelines.

### 2.1 Literature search

We searched all the literature (to 1 January, 2025) in the Medline (Ovid SP), Embase (Ovid SP) and Cochrane database, there was no language restriction. The involved key words included “SGLT2 is”, “SGLT-1/2 is”, “ESRD/diabetic nephropathy”, “diabetes mellitus” and “trials”; the detailed search strategy is accessible in **Supplementary Material 1**.

### 2.2 Inclusion and exclusion criteria

Inclusion criteria: 1) The study design was a RCT, or cohort study, or case-control study, or real-world study; 2) Adults participants with T2D; 3) SGLTis as the primary intervention; 4) Baseline measurements included estimated glomerular filtration rate (eGFR) and urinary albumin-creatinine ratio (UACR);5) participants without CKD; 6) Outcome variables encompassed CKD incidence or CKD-related event indicators.

Exclusion criteria: 1) Treatment duration shorter than 1 year; 2)T2D participants with CKD at baseline.

### 2.3 Definitions

PP of CKD was defined according to the Kidney Disease: Improving Global Outcomes (KDIGO) classification as prevention of incident CKD among individuals with baseline eGFR > 60mL/min per 1.73 m² and UACR < 30 mg/g (12). CKD events were defined as any of the following: 1) sustained decline in eGFR ≥ 40% or more, or doubling of serum creatinine; 2) progression to ESRD (dialysis or kidney transplantation); 3) CKD-related mortality; 4) progression of albuminuria to UACR ≥ 30mg/g.

### 2.4 Study selection and data extraction

**Figure 1:**
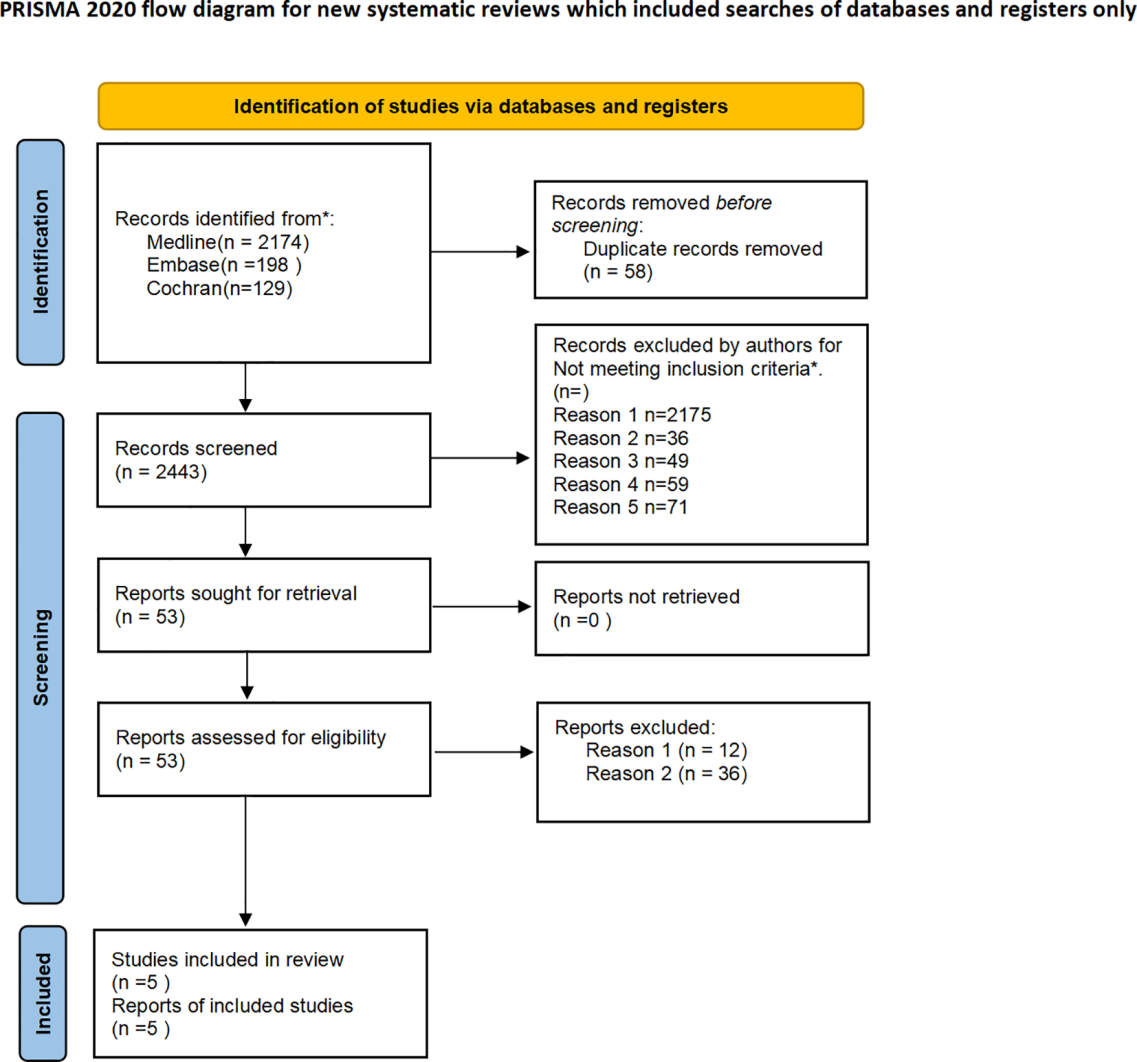
This study presents a flowchart. Reports identified through citation searches are positioned at the bottom to ensure chronological order. They underwent deduplication, title and summary screening, and full-text screening. Excluded research designs include non-randomized and crossover studies. Incorrect interventions involve SGLTis treatment for less than one year, while the incorrect population consists of non-type 2 diabetes patients and type 2 diabetes patients with chronic kidney disease.

Two authors carried out the course of screening and data collection independently. Of the 2501 clinical study articles retrieved, five RCTs were included in the analysis after removing 58 duplicates, reading the title, abstract, and full text, and screening using the above inclusion and exclusion criteria. See **Figure 1**. for the specific process of inclusion and exclusion of references.

### 2.5 Data collection

We extracted information about the participants characteristics, the regime and cases of intervention and control, the evaluating method, the follow-up time, and the study design from each study. We classified the trials into two categories, that is: 1) RCTs with kidney composite outcome in low-risk group, 2) observational studies with other outcomes.

### 2.6 Study evaluation and bias analysis

Two authors worked together to evaluate the bias of each study, using version 2 of the Cochrane tool for assessing risk of bias in randomized trial (RoB2).

### 2.7 Statistical analysis

The meta-analysis calculated the hazard ratio using revman software (5.3) and Mantel-Haenszel fixed-effect model, measured the degree of heterogeneity using I² statistics, and calculated 95% confidence intervals (95% CI).

## 3 Result

### 3.1 RCTs subgroup meta-analysis: SGLTis significantly reduce CKD-related outcomes in individuals with T2D who do not have CKD (CKD PP)

**Figure 2:**
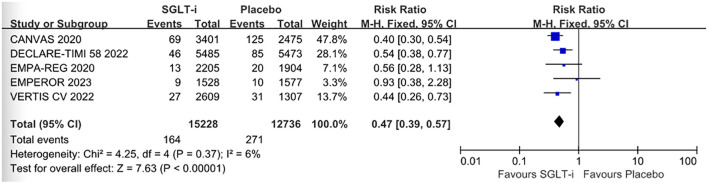
The forest plot shows the hazard ratio (RR) and 95% confidence interval (CI) of SGLT-i versus placebo in multiple studies. The X-axis is a logarithmic scale. The left side represents that SGLT-i is favorable, and the right side represents that placebo is favorable. The summary analysis results were represented by black diamonds. The overall hazard ratio (RR) was 0.47 (95%CI: 0.39, 0.57), suggesting that the risk of event occurrence in the SGLT-i group was significantly lower than that in the placebo group. The heterogeneity among the study populations was relatively low (I²=6%), and the Z-test (Z=7.63, P < 0.00001) indicated that the results were statistically significant. SGLT-i, a sodium-glucose coordinated transporter inhibitor. M-H, Mantel-Haenszel method. SGLTi classification: The intervention drug canagliflozin in 2020 of the CANVAS project is a dual inhibitor of SGLT-1/2, canagliflozin; The others are SGLT2 inhibitors.

**Figure 3:**
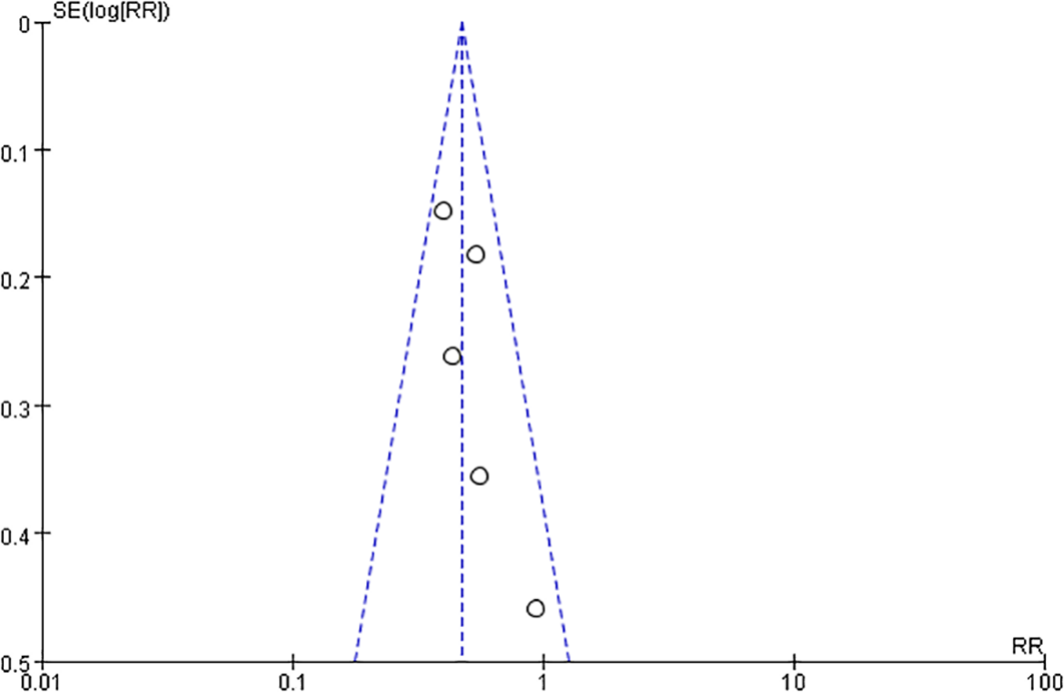
The funnel plot suggests that the publication bias in the meta-analysis is minimal. The X-axis shows the hazard ratio (RR), while the Y-axis displays the standard error of its logarithmic transformation (SE[log(RR)]). Each dot represents a study, and the blue dotted lines create a symmetrical funnel shape, indicating distribution within the 95% confidence interval. The research points are fairly evenly distributed within the funnel without significant leaning to one side, suggesting that publication bias is likely small.

The forest plot illustrates CKD outcomes for T2D patients without CKD (CKD PP), comparing SGLTis (empagliflozin, dapagliflozin, canagliflozin, ertugliflozin; n = 15,228) to a placebo group (n = 12,736), along with their respective 95% CIs. Participants receiving SGLTis had a mean follow-up duration of over 2 years. The overall RR for CKD-related outcomes was 0.47 (95% CI 0.39, 0.57), indicating a significantly reduced risk of CKD events in the SGLTi group compared to placebo (P < 0.0001). Heterogeneity among study populations was low (I² = 6%), and the Z test (Z = 7.63, *P* < 0.0001) supports generalizability to T2D populations without CKD or CKD PP crowd (**Figure 2**). The funnel plot shows most research sites within the funnel-shaped area and closely aligned with the central line, indicating symmetrical distribution and low risk of publication bias in included studies (**Figure 3**).

The meta-analysis of RCTs in the subgroup of non-CKD T2D population indicated that SGLTis were effective in reducing CKD outcome among the patients (PP, *P* < 0.0001). It indicated that they have the potential to significantly reduce the incidence of CKD in this population.

The details of the five eligible RCTs are presented in **Table 1** and described as follows.

**Table 1:** Information extracted from RCT^a^s.

Article information			Participants			Intervention	Control	Intervention period	Renal complex outcome definition	CKD PP Outcome	Reference
Author	Year	Journal	Characteristic	Mean age	Mean eGFR^b^ and UACR^c^ in CKD PP^d^ subgroups						
Adeera Levin	2020	Clinical journal of the American Society of Nephrology	Adults with T2D^e^, a BMI^f^ of<45kg/m2, established CVD^g^ , and eGFR≥30ml/min per 1.73 m2	61 years old	Mean eGFR = 83-84ml/min per 1.73 m2 Mean UACR = 7.1-8.0 mg/g	Total Patients (Empagliflozin 10mg/d, N = 2345 Empagliflozin 25mg/d) N = 2342; CKD PP subgroup (Empagliflozin 10 or 25mg/d) N = 2205	Total Patients (Placebo 10 or 25mg/d) N = 2333 CKD PP subgroup (Placebo 10 or 25mg/d) N = 1094	A Median follow-up of 2.6 years (interquartile range, 1.9-3.4)	Incident or worsening nephropathy (composite of progression to macroalbuminuria, doubling of serum creatinine accompanied by eGFR of ≤45 ml/min per 1.73 m2, initiation of KRT^h^, or death from kidney disease) and progression to macroalbuminuria.	The incidence of renal complex outcomes in the Empagliflozin group was lower than that in the placebo group (0.6% *vs.* 1.8%, HR 0.31, 95%CI 0.13, 0.63).	(13)
OfriMosenzon	2022	Diabetes Care	Patients with T2DM and either established multiple risk factors for ASCVD^i^ or current tobacco use	63.7 years old	Mean eGFR = 88.1ml/min per 1.73 m2 Mean UACR = 7.9mg/g	Total Patients (Dapagliflozin 10 mg/d) N = 8582 CKD PP subgroup (Dapagliflozin 10mg/d) N = 5485	Total Patients (Placebo 10mg/d)N = 8578 CKD PP Subgroup (Placebo 10mg/d N = 5473	A median follow-up of 4.2 years (interquartile range 3.9–4.4)	A sustained confirmed decrease by at least 40% of eGFR to eGFR <60 mL/min/1.73 m2, ESKD^j^ (defined as dialysis for ≥ 90 days, kidney transplantation, or sustained eGFR of <15 mL/min/1.73 m2), and/or kidney-related death.	The incidence of renal complex outcomes in the Dapagliflozin group was lower than that in the placebo group (0.8% *vs.* 1.6%, HR 0.54, 95%CI 0.38, 0.77).	(14)
BrendonL. Neuen	2022	American Journal of Kidney Diseases	Individuals with T2DM and eGFR ≥ 30mL/min/1.73 m2 who had or were at high risk for CVD	62.1 years old	Mean eGFR = 83.6ml/min per 1.73 m2 Mean UACR = 8.2mg/g	Total Patients (Canagliflozin) N = 5795 CKD PP subgroup (Canagliflozin) N = 3401	Total Patients (Canagliflozin) N = 4347 CKD PP subgroup (Placebo) N = 2475	A mean follow-up of 188.2 weeks	Sustained 40% decline in eGFR, kidney failure, or death due to kidney disease and sustained 40% decline in eGFR, kidney failure, or death, due to cardiovascular or kidney disease.	The incidence of renal complex outcomes in the Canagliflozin group was lower than that in the placebo group (2.1% *vs.* 4.9%, HR 0.40, 95%CI 0.25, 0.66).	(15)
David Z. I. Cherney	2021	Diabetologia	Individuals with T2DM and established ASCVD, with baseline eGFR ≥30 mL/min/1.73 m2	64.4 years old	Mean eGFR = 83.6ml/min per 1.73 m2 Mean UACR = 8.0mg/g	Total Patients (Ertugliflozin 5mg or 15mg/d) N = 5499 CKD PP subgroup (Ertugliflozin 5mg or 15mg/d) N = 2609	Total Patients (Placebo 5mg or 15mg/d) N = 2747 CKD PP subgroup (Placebo5mg or 15mg/d) N = 1307	A mean follow-up of of 3.5 years	Time to first occurrence of the composite of doubling of baseline serum creatinine, kidney dialysis/transplant or renal death.	The incidence of renal complex outcomes in the Ertugliflozin group was lower than that in the placebo group (1.0% *vs.*2.4%, HR 0.44, 95%CI 0.26, 0.73).	(16)
Javed Butler, MD, MPH, MBA	2023	JOURNAL OF THE AMERICAN COLLEGE OF CARDIOLOGY	Individuals with chronic HF^k^ and New York Heart Association functional class II-IV symptoms for at least 3 months	68.0 years old	Mean eGFR = 75.5ml/min per 1.73 m2 Mean UACR = 8.8mg/g	Total Patients (Empagliflozin 10 mg/d) N = 4860 CKD PP subgroup (Empagliflozin 10mg/d) N = 1528	Total Patients (Placebo10 mg/d) N = 4858 CKD PP subgroup (Placebo 10mg/d) N = 1577	A mean follow-up of 1.5 to 2.5 years	Sustained reduction of ≥50% eGFR or ESKD (chronic dialysis/renal transplant or sustained eGFR <15mL/min/1.73 m2 for patients with baseline eGFR ≥30 mL/min/1.73 m2 or sustained eGFR <10mL/min/1.73 m2.	The incidence of renal complex outcomes in the Empagliflozin group was lower than that in the placebo group (0.59% *vs.* 0.63%, HR 0.95, 95%CI 0.39, 2.34).	(17)

a.RCTs,randomized controlled trials; b.eGFR, estimated glomerular filtration rate; c.UACR,Urinary albumin/creatinine ratio; d.CKD PP,chronic kidney disease primary prevention; e.T2D, type 2 diabetes ; f.BMI,Body Mass Index; g.CVD, cardiovascular disease; h.KRT,Kidney Replacement Therapy; i.ASCVD, atherosclerotic cardiovascular disease; j,ESKD,End Stage Kidney Disease; k.HF, heart failure.

#### 3.1.1 EMPA-REG

A multinational RCT enrolled patients diagnosed with T2D, established atherosclerotic cardiovascular disease (ASCVD), and eGFR ≥ 30mL/min per 1.73 m². Participants were randomized to receive either empagliflozin (SGLT2i) or placebo. Among those meeting our study criteria, 2,205 patients were assigned to the empagliflozin group and 1,094 to the placebo group, with an average treatment duration of 2.6 years. Renal composite outcomes were compared between the two groups.

At baseline, there were no significant differences between the groups in terms of UACR, with values approximately 7.1 (4.4-14.1) mg/g in the placebo group and 8.0 (5.3-14.1) mg/g in the empagliflozin group, as well as eGFR, which was approximately 83.0 ± 16.0 mL/min per 1.73 m² in the placebo group and 84.0 ± 17.0 mL/min per 1.73 m² in the empagliflozin group. The incidence of renal composite outcomes was lower in the empagliflozin group compared to the placebo group (0.6% vs. 1.8%; HR = 0.31; 95% CI 0.16, 0.63). Additionally, the occurrence of UACR exceeding 300 mg/g was lower in the empagliflozin group than in the placebo group (3.2% vs. 3.6%; HR = 0.86; 95% CI 0.58, 1.27) (13). These findings suggest that empagliflozin may reduce the incidence of CKD in this patient population (without CKD at baseline).

#### 3.1.2 DECLARE-TIMI 58 trial

In a multinational RCT, patients with T2D and established cardiovascular or kidney disease were assigned to receive either dapagliflozin (SGLT2i) or placebo. Among those meeting our study criteria, 5,485 patients were allocated to the dapagliflozin group and 5,473 to the placebo group, with an average treatment duration of 4.2 years. The primary objective of this study was to compare renal composite outcomes between the two groups.

At baseline, there were no significant differences between the groups regarding UACR (approximately 7.9 (4.7-13.8) mg/g in the CKD PP group) and eGFR (approximately 88.1 ± 12.7 mL/min per 1.73 m² in the CKD PP group). The incidence of renal composite outcomes was lower in the dapagliflozin group compared to the placebo group (0.8% vs. 1.6%; HR = 0.54; 95% CI 0.38, 0.77) (14). These findings suggest that dapagliflozin may reduce the incidence of CKD in this patient population (without CKD at baseline).

#### 3.1.3 CANVAS

In this multinational RCT, participants with T2D, high cardiovascular risk, and eGFR ≥30 mL/min per 1.73 m² were treated with canagliflozin (SGLT1/2i) or placebo. Eligible patients for our analysis were randomly assigned to receive either canagliflozin (n = 3401) or placebo (n = 2475) for a mean treatment duration of 188.2 weeks. Serum creatinine levels collected during the study follow-up were measured centrally, and eGFR was calculated using the Modification of Diet in Renal Disease (MDRD) study equation to compare composite renal outcomes between the two groups.

Baseline UACR (approximately 8.2 (5.7-13.2) mg/g) and eGFR values (approximately 83.6 ± 16.4 mL/min per 1.73 m²) were similar between the two groups in CKD PP. The incidence of composite renal outcomes was lower in the canagliflozin group than in the placebo group (2.1% vs. 4.9%, HR = 0.40, 95% CI 0.25, 0.66) (15). These results indicate that canagliflozin may reduce CKD occurrence in this population without baseline CKD.

#### 3.1.4 VERTIS CV

Patients with T2D, ASCVD, and eGFR ≥30 mL/min per 1.73 m² were enrolled in this RCT to receive either ertugliflozin (SGLT2i) or placebo. Eligible patients for this analysis were randomly assigned to receive ertugliflozin (n = 2609) or placebo (n = 1307) for an average duration of 3.5 years, during which renal composite outcomes were compared between the two groups.

There were no significant differences in baseline UACR, with values approximately 8.0 (5.0-14.0) mg/g in the placebo group and 8.0 (4.0-14.0) mg/g in the ertugliflozin group, nor in eGFR, which was approximately 83.8 ± 16.4 mL/min per 1.73 m² in the placebo group and 83.5 ± 16.3 mL/min per 1.73 m² in the ertugliflozin group, between the two groups at baseline (eGFR and UACR were eligible). The incidence of renal composite outcomes was significantly lower in the ertugliflozin group compared to the placebo group (1.0% vs. 2.4%, HR = 0.44, 95% CI 0.26, 0.73) (16). These findings indicate that ertugliflozin may decrease the occurrence of CKD within this population (without CKD at baseline).

#### 3.1.5 EMPEROR

In a multinational RCT, patients with T2D, chronic heart failure, and New York Heart Association (NYHA) class II–IV symptoms persisting for at least three months were assigned to receive either empagliflozin (SGLT2i) or placebo. Among those meeting our study criteria, 1,528 patients were allocated to the empagliflozin group and 1,577 to the placebo group, with an average treatment duration ranging from 1.5 to 2.5 years. The study compared renal composite outcomes between the two groups.

At baseline, there were no significant differences between the groups regarding UACR (approximately 8.8 (5.3-15.9) mg/g in CKD PP) and eGFR (approximately 75.5 (67.5-86.5) mL/min per 1.73 m² in CKD PP). The incidence of renal composite outcomes was comparable between the empagliflozin and placebo groups (0.59% vs. 0.63%; HR = 0.95; 95% CI 0.39, 2.34). These findings suggest that empagliflozin may not have a significant impact on reducing the incidence of chronic kidney disease in this patient population. A major limitation of this trial was the relatively short duration of double-blind treatment, which constrained our ability to correlate changes in eGFR with the occurrence of major adverse renal outcomes (17).

Although the baseline characteristics, duration of intervention, and CKD outcomes varied among participants in the five RCTs, SGLTis exhibited a consistent bias towards primary prevention in the CKD subgroup without T2D. By integrating the findings from these studies along with meta-analysis results, we propose that treatment with SGLTis may reduce the incidence of renal composite outcomes in T2D patients who do not have CKD, thereby potentially preventing the onset of CKD.

### 3.2 Observational studies analysis: in the T2D population without CKD (CKD PP), SGLTis can significantly reduce the occurrence of CKD compared with the control hypoglycemic drugs

In 2021, a real-world study enrolled patients with T2D who were treated with SGLTis and other hypoglycemic agents (OHGLAs), with follow-up extending beyond 12 months. The study included 11,321 patients receiving SGLTis (dapagliflozin or empagliflozin) and 42,077 patients on OHGLAs, which comprised dipeptidyl peptidase-4 inhibitors (DPP4i), metformin, glucagon-like peptide-1 (GLP-1), insulin, sulfonylureas, or meglitinides. Among those who met the inclusion criteria for our analysis, there were 5,832 patients in the SGLTis group and 5,936 patients in the OHGLAs group. The median follow-up duration was 1.7 years. When compared to the control group, renal-specific outcomes in the SGLTis cohort demonstrated a significant reduction (0.6% vs. 1.0%, HR = 0.62; 95% CI 0.45, 0.86), although the P value was not reported in the original article (18). Furthermore, when comparing SGLTis to OHGLAs (non-SGLTi), it was observed that treatment with dapagliflozin or empagliflozin significantly decreased the incidence of CKD among T2D patients without pre-existing CKD.

In 2023, a real-world study enrolled T2D patients taking SGLTis and DPP4is, followed for more than 12 months. The study included 16,065 SGLTis (dapagliflozin or empagliflozin) and 23,208 DPP4is (sitagliptin, linagliptin, vildagliptin or saxagliptin). Patients who met our criteria were 5,161 patients in the SGLTis group and 5,306 in the DPP4is group. The median follow-up was 38 months. Compared with the control group, renal specific outcomes in the SGLTis group (HR = 0.77, 95% CI 0.61, 0.97) were significantly reduced in the SGLTis group (*P* = 0.03) (19). These results indicate that SGLTis (dapagliflozin, empagliflozin) can significantly reduce the incidence of CKD in T2D population without CKD compared with DPP4is (CKD PP).

Due to discrepancies in renal outcome measures between the two observational studies and the five aforementioned RCTs, a meta-analysis was not performed (see **Table 2**). Nevertheless, the findings from these observational studies are consistent with the conclusions derived from the meta-analysis: in patients with T2D who do not have CKD (CKD PP), treatment with SGLTis (n = 10,993; all participants had follow-up durations exceeding one year) is associated with a reduced risk of adverse renal outcomes. This suggests a preventive effect against the development of CKD.

**Table 2:** Information extracted from observational studies.

Article information			Participants		Study design (group)	Follow-up duration	Renal outcome definition	CKD PP outcome	Reference
Author	Year	Journal	Characteristic	The mean eGFR^a^ and UACR^b^ of CKD PP^c^ subgroup					
Meir Schechter	2021	Cardiovascular Diabetology	Patients with type 2 diabetes, and 57.0% with diabetes duration longer than 10 years. The mean age was 62.4 years. Most of them overweight.	Mean eGFR = 88.3ml/min per 1.73 m2 Median UACR = 13mg/g	11,321 SGLTi^d^ initiators *v*s 42,077 oGLAs^e^ initiators. Non-CKD were eligible CKD PP subgroup: SGLTis (Dapagliflozin or Empagliflozin) N = 5832; OGLAs (DPP4i^f^, Metformin, GLP-1 ^g^, Insulin, Sulfonylurea or Meglitinides) N = 5936	A median follow-up of 1.7 years	The specific renal outcome included new ESKD^h^ or ≥ 40% reduction in eGFR.	Initiation of SGLTis was associated with a lower risk for the specific renal outcome (0.6% *vs.* 1.0%, HR 0.62, 95%CI 0.45, 0.86).	(18)
Cheli Melzer Cohen	2023	Clinical Journal of the American Society of Nephrology	Patients with type 2 diabetes. The mean age was 61 years. 10,467 patients had no evidence of cardiovascular or kidney disease.	Mean eGFR = 90ml/min per 1.73 m2; Median UACR=12mg/g	16,065 vs 23,208 Patients initiated SGLTis *v*s DPP4is. Non-CKD were eligible. CKD PP subgroup: SGLTis (Empagliflozin or Dapagliflozin) N = 5161; DPP4is (Sitagliptin, Linagliptin, Vildagliptin or Saxagliptin) N = 5306	A median follow-up of 38 months.	The specific renal outcome Included confirmed≥40% decline in eGFR or kidney failure.	Initiation of SGLTis was associated with a lower risk for the specific renal outcome (HR 0.67, 95%CI 0.44, 1.02).	(19)

a. eGFR, estimated glomerular filtration rate; b.UACR,Urinary albumin/creatinine ratio; c.CKD PP,chronic kidney disease primary prevention d.SGLTi, sodium/glucose cotransporter inhibitor; e. oGLAs, other glucose lowering agents; f. DPP4is, dipeptidyl peptidase 4 inhibitors; g.GLP-1, glucagon-like peptide-1; h.ESKD, end stage kidney disease.

Integrating the findings from the aforementioned RCTs and real-world observational studies, we conclude that SGLTis provide primary preventive benefits against CKD in patients with T2D without pre-existing CKD. Both subgroup analyses from RCTs and data from observational studies consistently demonstrate that SGLTis reduce the risk of adverse renal outcomes in this patient population, supporting their role in the primary prevention of CKD.

### 3.3 Risk of bias

**Figure 4:**
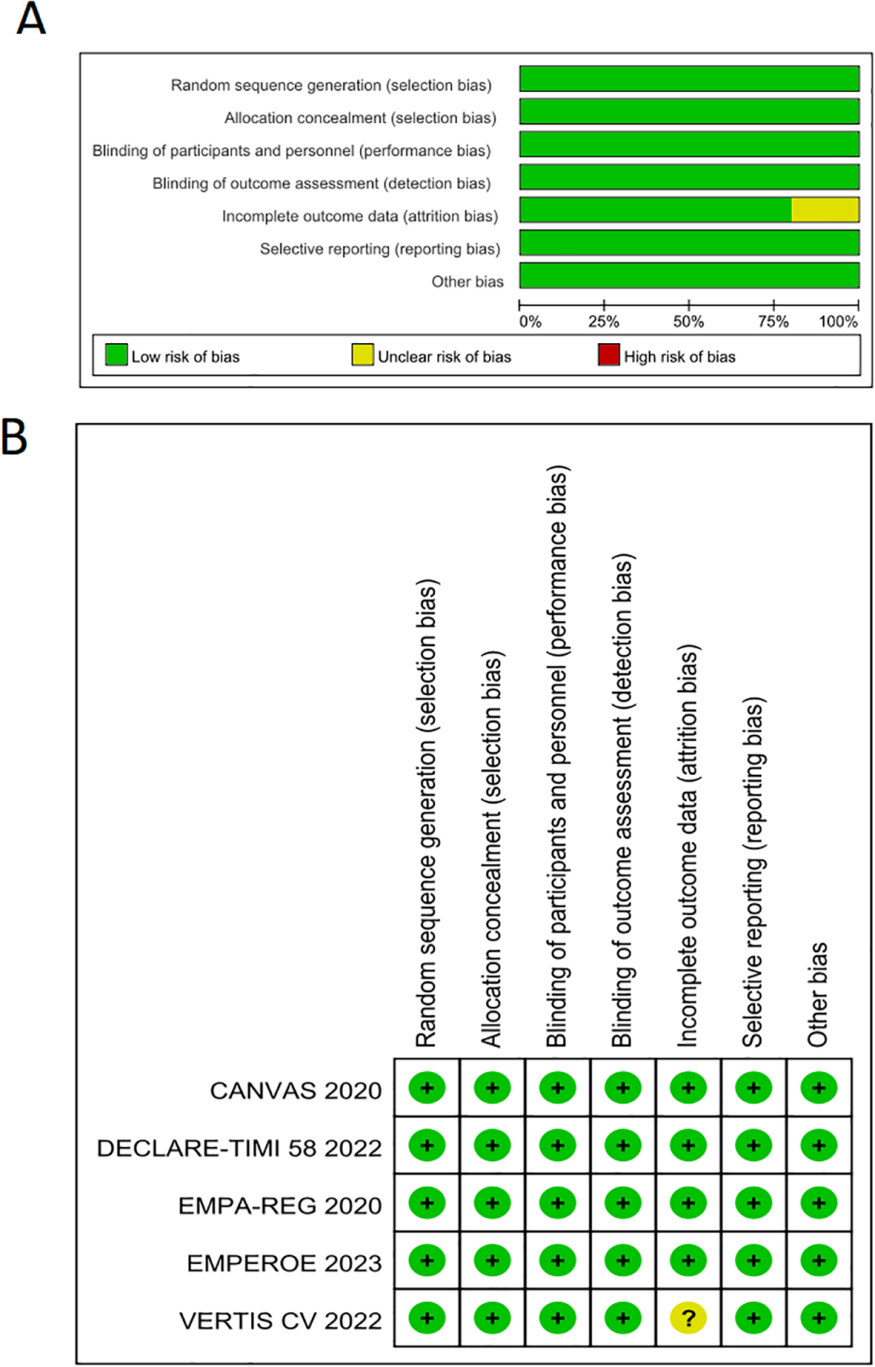
Risk of bias (using ROB2 for RCTs ). **(A)** Risk of bias graph for RCTs: authors’judgements about each risk of bias item presented as percentages across all included RCTs. **(B)** Risk of bias summary for RCTs: authors’ judgements about each risk of bias item for each included RCTs.

The risk of bias assessments for each study are summarized in **Figure 4**.

The risk of bias assessments for the five randomized controlled trials (RCTs) are summarized in **Figure 4**. All five RCTs employed randomized, double-blind methodologies, and their outcome measures (kidney composite endpoints) were objectively quantified, minimizing subjective influence from participants and resulting in a low risk of bias in this aspect. Regarding attrition, the fourth RCT exhibited a slightly higher loss to follow-up rate (approximately 12–13%), with specific information about these participants unavailable. The remaining RCTs had lower attrition rates (< 10%); however, they did not include data from these participants in the final analyses. Consequently, there are some concerns related to deviations from intended interventions. Overall, the quality of these RCTs is high. Although some concerns exist, the large sample sizes, extended follow-up periods, and relatively low attrition rates suggest that these issues are unlikely to significantly impact the study outcomes.

## 4 Discussion

This systematic review and meta-analysis provides evidence that SGLTis (mainly SGLT2i, including SGLT1/2i) are effective in preventing the occurrence of CKD in patients with T2D who have not yet developed CKD. Across five RCTs and two large real-world studies, treatment with SGLTis was consistently associated with lower rates of CKD-related composite outcomes, supporting their role in primary prevention.

In addition to the five RCTs included in our analysis, we identified several other studies on SGLTis for T2D complicated by cardio-renal disease during the screening process. These studies have also been extensively cited in the literature. However, upon thorough examination of their full texts, we discovered that some of these studies did not include renal composite outcomes among their reported indicators (20); Some study groups, regardless of the presence or absence of T2D (21).; Some studies primarily focused on investigating the efficacy of SGLTi in the context of heart failure (22).; Some follow-up periods were insufficient to adequately assess the renal composite outcome. Additionally, certain studies did not analyze kidney outcomes within the low-risk group, nor did they provide statistical inference on primary prevention (23). These limitations rendered them incompatible with the inclusion criteria and objectives of this review. Consequently, we opted not to include these seemingly relevant studies.

A key point of our analysis is that while SGLTis are known to significantly reduce the risk of renal composite outcomes in patients with T2D and CKD, once CKD has already developed, its progression cannot currently be halted. If it can be demonstrated that SGLTis can decrease the incidence of CKD or CKD-related events in individuals with T2D who do not yet have CKD (PP), this evidence would provide a valuable new strategy for reducing the occurrence of CKD within this population.

Our meta-analysis of five RCT subgroups revealed a 47% reduction in the risk of renal outcomes for CKD (RR = 0.47; 95% CI 0.39, 0.57), which represents a clinically significant finding. This conclusion aligns with real-world data, suggesting that our results are robust and warrant high confidence.

The protective mechanisms of SGLTis in the kidney are likely to be multifactorial. In addition to their glucose-lowering effects, these agents reduce intraglomerular pressure through the modulation of tubuloglomerular feedback, enhance blood pressure control, and decrease albuminuria. Furthermore, additional benefits such as reduced renal hypoxia and anti-inflammatory effects may also play a significant role in their overall efficacy (24–26).

Limitation of our research: There are several important considerations. First, while the date are compelling, not all trials use consistent measures for kidney outcomes, which may hinder direct comparisons. Additionally, the relatively short follow-up periods in some studies, such as the EMPEROR trial, limit our ability to assess the long-term effects of SGLTis on CKD prevention fully. Furthermore, the ideal study population for CKD prevention is patients with an eGFR greater than 90 mL/min per 1.73 m²; however, these individuals typically have good renal function and limited opportunities for early monitoring. The lengthy observation required for outcome assessment and infrequent inclusion in clinical studies further restricts available information on this group. Consequently, our conclusions may be limited.

Despite these limitations, our findings carry significant clinical implications. CKD is largely irreversible once established, and current treatment options remain limited. The early initiation of SGLTis in patients with T2D who do not have CKD (PP) may represent a novel strategy to delay or prevent the onset of the disease, potentially offering long-term benefits in reducing ESRD, cardiovascular events, and healthcare burdens.

## 5 Conclusion

In conclusion, the findings from this meta-analysis and systematic review provide robust evidence supporting the use of SGLTis as an effective strategy for the pp of CKD in patients with T2D. Given their potential to delay or prevent the progression of kidney disease in high-risk populations, this intervention demonstrates both clinical efficacy and cost-effectiveness. Nevertheless, further RCTs are necessary to validate our conclusions.

## Data Availability

The datasets presented in this study can be found in online repositories. The names of the repository/repositories and accession number(s) can be found in the article/**Supplementary Material**.
